# “Who’s got the infants in mind?” A qualitative exploration of the barriers and enablers to commissioning parent-infant relationship services in England

**DOI:** 10.1186/s12913-025-13215-5

**Published:** 2025-08-02

**Authors:** Lorraine McSweeney, Karen Bateson, Wook Hamilton, Bronia Arnott

**Affiliations:** 1 Population Health Sciences, Newcastle University, Newcastle upon Tyne, NE2 4HH UK; 2 Oxford Parent-Infant Project, Kidlington Centre, Oxford, OX5 2DL UK; 3 Parent-Infant Foundation, Office 7, 35-37 Ludgate Hill, London, EC4M 7JN UK

**Keywords:** Qualitative, COM-B, Commissioning, Evidence-use, Parent-infant relationships, Infant mental health.

## Abstract

**Background:**

The earliest relationships between babies and their caregivers can act as a risk factor for social and emotional wellbeing in infancy and impact on later development. Difficulties in parent-infant relationships (PAIR) are characterised by inequalities, with families experiencing adversity at greater risk. In the absence of support, these relationship problems may require later, more expensive services. Interventions exist to address PAIR difficulties but are not widely commissioned, with regional disparities, and infants being under-served. This project qualitatively explored barriers and enablers to commissioning PAIR services.

**Methods:**

Individuals with commissioning responsibilities relating to PAIR working in Clinical Commissioning Groups or Local Authorities in the North East North Cumbria (NENC) region were invited to participate. Qualitative semi-structured interviews were informed by a topic guide developed through practitioner involvement. Nine interviews were completed online between February and April 2022. Anonymised transcripts were analysed using Framework Analysis.

**Results:**

Barriers and enablers to commissioning were general, specific to PAIR services, or related to the pandemic context. General factors included the nature of the *commissioning process*, the importance of taking a c*ollaborative approach to commissioning* and the constraints of *funding processes*. Commissioners valued being able to demonstrate impact and value for money through s*ervice evaluation* but faced challenges in doing so. Specific barriers relating to PAIR commissioning included a lack of awareness of the importance of *infant mental health* although there was an acknowledgement of the importance of *maternal and parent-infant services/support* during the early years. Factors amenable to change were mapped on to the Behaviour Change Wheel to inform the future co-development of a resource to address barriers and enablers.

**Conclusions:**

This paper is the first to report a pragmatic, applied qualitative exploration of barriers and enablers to commissioning parent-infant relationship services. Insights from commissioners with a wide range of portfolios identified key factors operating at the individual level which were amenable to change. In addition, there were barriers beyond the individual level, such as funding. Using a pragmatic approach, we identified best-fit behaviour change initiatives to develop a commissioning support toolkit to increase access to support, improve outcomes, and decrease inequalities, addressing infants as an under-served group.

**Supplementary Information:**

The online version contains supplementary material available at 10.1186/s12913-025-13215-5.

## Background

Infant mental health is the social and emotional wellbeing of babies and the quality of relationship between a baby and its parent(s) is a strongly protective factor for current and future mental health. Research suggests that around one-third of babies experience mild-moderate parent-infant relationship (PAIR) difficulties but a further 15% experience significant difficulties which risk seriously disrupting multiple aspects of development [1]. There is also evidence that rates can be higher in families facing adversity (such as child maltreatment, maternal substance use) [1] with families with multiple factors at highest risk. The UK government in their guidance on the Family Hubs and Start for Life programme [2] uses infant attachment quality as a proxy measure for PAIR difficulties and estimates the prevalence of ‘disorganised attachment’ at 10% of the population. This is the most vulnerable group and without specialised, early help, relationship problems may require later, more expensive interventions including, in severe cases, a child being taken into care. Professionals working with babies also recommend supporting those with other types of ‘insecure attachment’ as they also experience distress which can impact their development negatively. Additionally, there is merit in services that work to prevent PAIR difficulties from developing. Interventions to address PAIR difficulties exist [3–5] but are not widely commissioned. Whilst different professions/services can support PAIR, specialist therapeutic interventions tend to be offered through mental health services, yet these are sparsely available. The Rare Jewels Report in 2019, found in England and Wales 42% of Clinical Commissioning Groups (CCGs) made no mental health provision for under 2s [6]. Although the number of PAIR services has grown from 27 to 46 [7, 8] in the intervening years many families in need still have no access to support. In particular, there are geographical inequalities in access, with only one PAIR service in the North East North Cumbria region [8]. PAIR services are one of the funded areas as part of Family Hubs and Start for Life provision, with £100m funds dedicated to perinatal mental health and PAIR [9]. This is therefore an important and timely exemplar for considering barriers and enablers to commissioning.

Commissioning is a process ‘for deciding how to use the total resource available for families in order to improve outcomes in the most efficient, effective, equitable and sustainable way’ [10] and is a model that has been adopted by local areas in England to improve outcomes and address budget pressures. This is increasingly relevant when finances are under strain and demand on services is rising [11]. Commissioning is the ‘continual process of planning, agreeing and monitoring services. Commissioning is not one action but many, ranging from the health-needs assessment for a population, through the clinically based design of patient pathways, to service specification and contract negotiation or procurement, with continuous quality assessment’ [12]. Commissioning processes can vary but are frequently underpinned by the same fundamental cycle: understand, plan, do, review. This process starts with identifying local needs, listening to family’s views, and reviewing existing provision. The next phase involves strategically considering the most optimal way of addressing identified needs. Next, investment decisions are made, and services procured. Finally, delivery is monitored and desired outcomes measured.

Those involved in the commissioning process, use a range of information to try and build a convincing, cohesive and persuasive case to ‘navigate a way through the system, justify decisions and, convince others to approve and/or follow the suggested course’ [13]. A principle of good practice in commissioning is basing decisions on available evidence about the impact of services on outcomes and their value for money. The difficulties of the use of evidence from research in the commissioning process has been highlighted by van der Graaf et al., (2018) [14] concluding that for research to be used by commissioners it needs to be actively mobilised.

We can therefore view commissioning as a process of making decisions and taking action informed by knowledge sources, including evidence. This process can be considered through the lens of behavioural science and the barriers and enablers to change identified. The Capability, Opportunity, Motivation and Behaviour Model (COM-B) [15], which is widely used to conceptualise individual level change, can be applied as a theoretical framework to understand commissioning. The COM-B model is a framework for understanding behaviour and proposes that behaviours can occur when the individual has the capability, opportunity and motivation to take action. Once barriers and enablers are understood the Behaviour Change Wheel (BCW) [15] can be employed to suggest intervention functions to addresses these individual level levers.

In July 2022, a more ‘place-based approach to commissioning’ was created through the legal establishment of Integrated Care Systems (ICSs) (previously CCGs) [16] through the Health and Care Act 2022. ICSs bring together NHS, local authority and third sector bodies to take on responsibility for the resources and health of an area or ‘system’. Greater integration between services for children and their families across sectors has also been facilitated in England through the Family Hubs programme [17] mirroring the ICS transformation by joining up across the system. The £300m Family Hubs and Start for Life investment provides a unique opportunity for joint commissioning of services across the ICS partnership as part of the core offer.

There is limited evidence to date relating to the commissioning of PAIR services. Work by Homonchuk & Barlow [18] suggests that factors influencing commissioning may include opportunity for long-term cost reduction and the potential to embed within existing services. Further work is required to understand factors affecting decision making and what evidence would be convincing as part of that process.

This pragmatic and applied project explored barriers and facilitators to commissioning PAIR services in the North East North Cumbria (NENC) region using an innovative approach employing the COM-B model and the BCW to inform the future co-development with commissioners of a resource to support their decision making and action. By engaging end-user audiences through knowledge mobilisation at an early stage as recommended by Waddell and colleagues [19] this research will inform commissioning in local areas in England with the aim of increasing access to support, improving outcomes, and decreasing inequalities for families.

## Methods

### Study design, setting and participants

A scoping exercise was previously conducted by the authors to identify those in the NENC region working in CCGs or Local Authorities involved in decision making around parent-infant relationship or infant mental health services. The scoping exercise used publicly available information about job titles, to identify individuals working in the region who had the potential to commission PAIR services as part of their role. Using purposive recruitment methods, those identified by the scoping exercise were invited by email to participate in qualitative semi-structured interviews lasting around 30–60 min to explore and understand barriers and facilitators to commissioning PAIR services. Potential respondents were also recruited through the NHS North of England Commissioning Support Unit and commissioner forum groups by sharing the study information and researcher contact details for those interested to respond. This was not a clinical trial therefore a clinical trial number was not applicable.

### Data collection

An interview topic guide was developed with a practice involvement group, consisting of four commissioners working in other regions who reviewed drafts and engaged in pilot interviews. The topic guide (Supplementary material 1) included questions on commissioners’ current role and remit; experiences and knowledge of infant mental health and PAIR; and facilitators and barriers to commissioning services.

Participants who agreed to be interviewed were sent the study information sheet and asked to complete an e-consent form. All interviews took place remotely due to the Covid-19 pandemic restrictions in place at the time. Thirteen commissioners responded to the email/information invite, eleven participants consented to be interviewed, two respondents were unable to complete their planned interview, however, data saturation, where no new information was gathered, was reached after nine interviews which took place between February and April 2022. The one-to-one interviews were conducted by Research Associate, LM using the Microsoft Teams platform and with participants’ permission, the video and transcription function enabled. LM explained their role as a researcher who had little knowledge of commissioning processes. Additional notes were made after the interview and transcriptions were checked for accuracy by a research team member and anonymised and the recordings deleted.

### Data analysis

Data were repeatedly read and coded independently by LM using the qualitative software NVivo to aid coding [20]. Codes were grouped together into categories and analysed using Framework Analysis [21] this includes: (1) Data familiarisation, (2) Coding (inductive coding was used), (3) Categorisation, (4) Development of the framework, (5) Charting data into the framework and, (6) Interpretation of data to develop themes [22]. To minimise researcher bias, codes and themes were discussed with the project lead (BA) and the wider study team (KB, WH). Using a framework method of verbatim quotes allowed for transparency of coding.

**Fig. 1:**
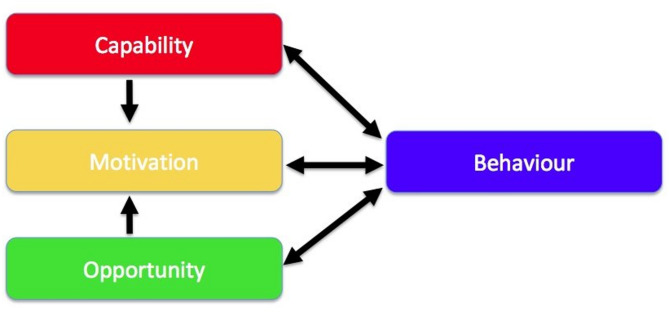
The COM-B system—a framework for understanding behaviour [15]

Following interview analysis and to identify where behaviour change may be applied in the commissioning of PAIR services, the COM-B model [15] was utilised to explore barriers and enablers and to inform a future commissioning support resource. The COM-B model is a framework for understanding behaviour and proposes that a particular behaviour will occur only when the person concerned has the capability and opportunity to engage in the behaviour and is more motivated to enact that behaviour more than any other behaviours’ [23] (Fig. 1).

### Mapping the interview findings onto the COM-B model

Whilst the COM-B components analysis highlighted both individual and non-individual level barriers and facilitators, a pragmatic decision was made to focus on the individual level as this was the initial aim and scope of the project. However, there was recognition that some non-individual level levers could potentially be addressed through a commissioning support tool. Further, for the individual level barriers there would be different ways of addressing these, as suggested by the BCW [15]. Therefore, a pragmatic decision was required to focus on the areas that could realistically, given the available time, resources and control, be implemented for change.

### Behaviour change wheel

**Fig. 2:**
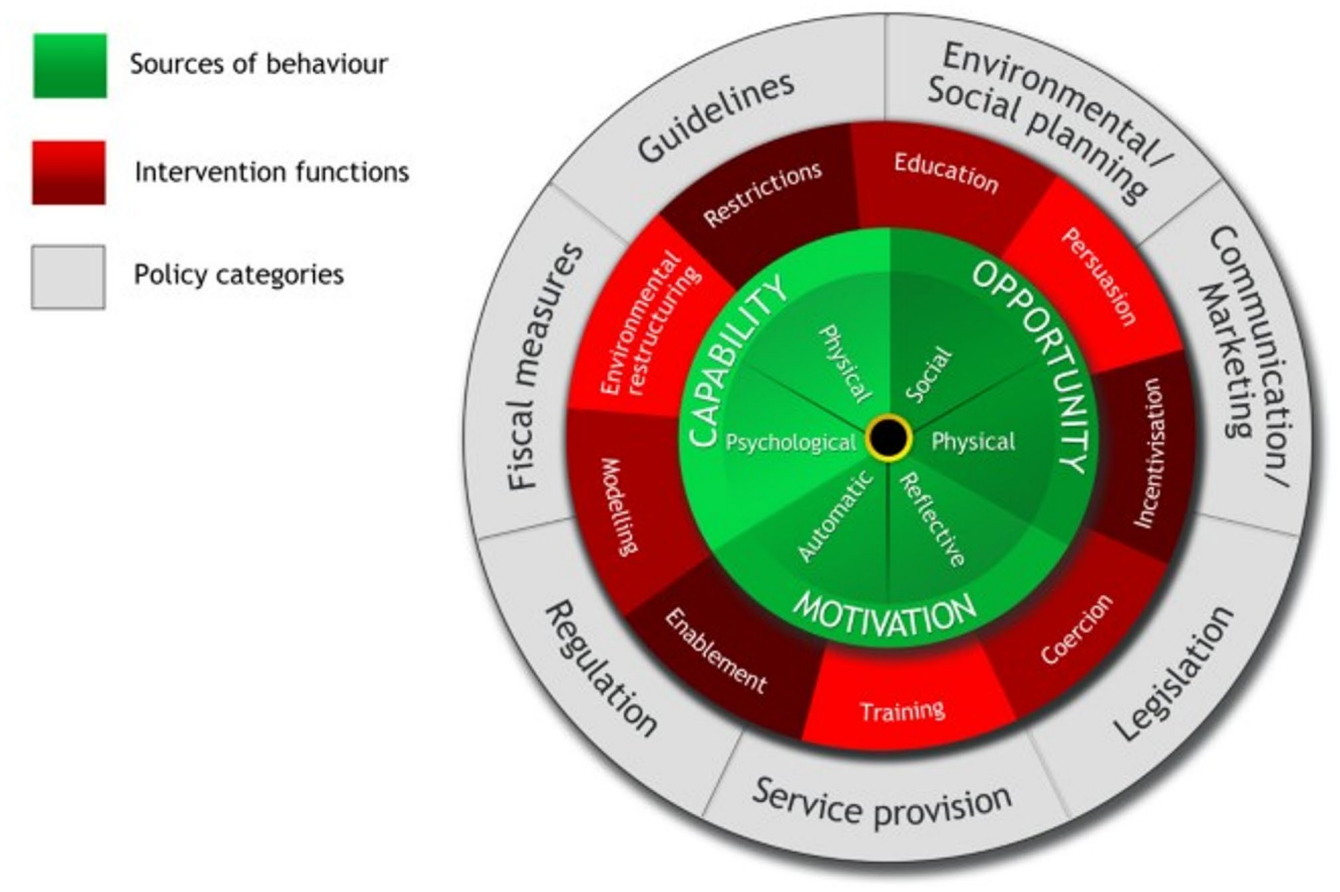
The behaviour change wheel [25]

The BCW consists of three layers (Fig. 2). The hub of the wheel (The COM-B model) identifies the sources of the behaviour that could prove fruitful targets for intervention. Surrounding the COM-B model is a layer of nine intervention functions. Then the outer layer identifies seven types of policy categories [24] and this was used to identify ways to impact commissioning behaviours.

## Results

Nine semi-structured interviews were conducted with commissioners employed by the Local Authority or NHS CCG (replaced with ICSs in July 2022). The portfolios held by the commissioners varied, including responsibilities for children and young people, maternal, perinatal, mental health, and several had overall responsibility of all mental health services from birth to death. Portfolios also included children and young people with special educational needs, disabilities and learning disabilities. No further information/characteristics about the interviewees has been shared to protect participant confidentiality. Seven themes were identified from the data:

- The commissioning process.
- Collaborative approach to commissioning.
- Funding process.
- Service impact and evaluation.
- Importance of infant mental health.
- Maternal and parent-infant services/support.
- Impact of Covid-19 on commissioning.

Whilst there were barriers and facilitators experienced that could be common across all commissioning areas, the findings also highlight issues pertinent to the commissioning of PAIR. The findings are presented by theme and are illustrated with anonymised participant quotes.

### The commissioning process

The commissioners reported that their ultimate aim was to reduce health inequalities at the earliest point in life. Their interests also included making sure children had the best start in life, ensuring families with young children received support when required, and that outcomes for families were improved.

*“So we’ve got obviously a lot of ambitions around the work that we do*,* but ultimately we are here to reduce health inequalities through the earliest possible point of life. So that’s what we aim to do” (ID: P1).*

*“…my best outcome I suppose would be around the fact of*,* I don’t necessarily have to be commissioning the more higher-level services because we’re getting in there earlier. So that would be my lifelong aspiration if I’m gonna [sic] be honest” (ID: P7).*

The commissioners recognised that the new changes from CCGs to ICSs may be a facilitator. It was hoped that:

- The new set ups would provide a co-ordinated process with collaborative working and pooled resources.
- There would be more focus on ‘place-based’ approaches which would be of benefit. ‘Place-based’ areas have clear mental health guidance.
- A ‘systems’ approach may allow for commissioning of services on a longer-term basis.

However, there was some concern that some small communities might get ‘lost in the process’ under new arrangements.

*“I think the biggest risk of that change is a place can get lost*,* so very small communities might get lost in the noise. I worry about what it means for the equality agenda overall because we don’t have the populations that*,* that equally reflect each other in that conversation” (ID: P5).*

The size of communities could relate either to their geographical size or the size of their population, in relation to other communities within the same region. There was a concern that the interests of smaller communities may be better represented by the current model of more local commissioning and that under new arrangements commissioning may be influenced by larger and/or more populated areas.

Commissioners felt that the sharing of information and ideas with other commissioners was valuable, and some had the opportunity to engage in weekly/monthly online meetings and forums with others.

*“We now do those [meetings] online*,* they’re every Tuesday afternoon for about 45 minutes*,* so they’re more regular but shorter. And I think what we do now is we discuss a lot more of what’s coming down the line. So*,* there’s a policy coming out next week– what*,* what do we need to do around this? What’s happening? And it just helps us all connect. I don’t think it’s yet enabled us to commission things at any sensible scale or across a region*,* but I think that time is coming…” (ID: P5).*

They emphasised that these meetings were useful for general discussion of issues relating to their broad portfolios. It was reported that having the opportunity to have ‘honest conversations’ and ‘peer mentoring’ was helpful, enabling participants to build up expertise and relationships. However, one criticism was that the forums tended to be attended by the same core of people limiting opportunities to learn from wider perspectives/experiences. Specifically, they were not spaces where they could hear from others who had commissioned PAIR services and therefore to understand best practice in this area. They reported that they would benefit from being able to hear about what had worked well and what people would have done differently from those with experience.

#### Collaborative approach to commissioning

Relationships were highlighted as an important aspect in commissioning. Commissioners reported that the process should involve a wide range of people and groups from the beginning of planning for a service. It was stated to be essential that commissioners know their local area and communities; understanding the local population needs was a key enabler. It was highlighted that the commissioning process had got better at engaging with families and communities; commissioners now dedicated a lot of time to this, promoting feelings of ownership in their local areas.

*“You can’t build the service unless you’ve got the families there. And obviously the infants are a bit too little to say*,* but I’m sure there’s data and I’m sure there’s insight that could be shared on behalf of those infants*,* and then the spokespeople of the parents*,* to develop those services… you should have representation from every group and even the ones that you think that might not be interested quite often are. You know*,* bit left field*,* so like your charitable organisations” (ID: P1).*

The types of professionals who commissioners felt it was important to involve in the process when considering services relevant to PAIR and infant mental health included: clinicians/GPs/health practitioners; midwives/health visitors/maternity services; social workers; service providers; childminders and nurseries; councillors; and the voluntary sector. In addition, commissioners reported that in recent years there had been positive changes with better working relationships between commissioners and service providers, which facilitated the commissioning process.

#### Funding process

Commissioners spoke about their spending being constrained by budgets. Commissioners have an obligation to realise government identified aims for example the ‘NHS Long Term Plan’ [26] outlines specific areas of need determining the types of services that funding should be used for. However, it was highlighted that although these directives ensure national priorities are addressed at a local level, there may be other services required in the local area without any budget available.

*“I mean fairly obviously value for money is always a consideration because it’s public money when you’re commissioning the provision. Uhm. I think it’s*,* some of it’s a challenge because as I said*,* with the Long Term Plan now a lot of it is actually dictated how much we have to spend on mental health provision. So we might not necessarily agree with the trajectories in relation to the Long Term Plan*,* but there’s an expectation that we do commission it to that level. Uhm. Yeah. But I think a lot of it is in in relation to sort of the cost effectiveness*,* value for money*,* it’s about impact*,* isn’t it as well? So it’s thinking about if you’re looking at value for money*,* is actually that provision doing what it says on the tin’? Is it delivering what we’re expecting it to do for the money that we’re investing?” (ID: P2).*

Additionally, funding from central government for a particular type of service often will only be provided on a short-term basis, and this was reported as a barrier for commissioners. Even if services have shown benefit and value, commissioners then must find funding from other sources, as they perceived providing a service to their communities which cannot be sustained for their long-term benefit to be unethical. Without sustainable funding a PAIR service may have to be withdrawn. Funding uncertainty can also make staff recruitment difficult. Commissioners also highlighted that that in some cases there may be a shortage of a skilled workforce, in professions such as health visiting, which may mean that even when funding is available there are challenges to spending the money appropriately due to workforce gaps as staff seek employment security.

*“If you*,* if you’ve got a workforce who’s constantly wondering whether they’re gonna [sic] have a job next year…They’re probably not gonna be 100% onboard. So we try not to do that [short-term contracts]. And then monies like from the local authorities*,* we tend to try– the short term we tend to try and use as an enhancement*,* or is there a short bit that we need to do to get to the next step? And that side of things. So that bridging element. So that’s what we tend to try and use the shorter term money for” (ID: P7).*

#### Service impact and evaluation

The importance of being able to demonstrate impact and value for money was identified by commissioners, however it was also acknowledged that doing so could be challenging. Measures for recording impact included: improvement activity, patient/user outcome measures, workforce development measures, and whether the service was demonstrating value for money. Most providers/services were expected to deliver key performance indicators and were contracted to do so.

*“I would expect a month- to get a monthly report and activity in terms of improvements*,* you know*,* patient outcome measures*,* uhm workforce development measures*,* where they’ve delivered*,* yeah*,* what they’re*,* what the KPIs [key performance indicators] are…uhm and*,* and yes*,* some inbuilt evaluation over a period of a year or a set time*,* so we can actually see the impact and whether it’s value for money” (ID: P3).*

Whilst it was reported as important to collect quantitative data when considering impact, of most interest and value was service users’ feedback and case studies (quoted by one interviewee as ‘75% of focus’). Including service users’ voices and listening to their concerns was desirable, but for some, not always achievable.

*“I wouldn’t just expect it from the provider– if you could ideally have it*,* I’d want observations from places like health visitors or parent groups or schools or*,* or whatever. So you could actually try to get that*,* actually we’ve seen a real mum feels really comfortable with baby*,* baby’s settling more. You can see*,* or if they’re a bit older*,* you know you can see that the child’s got a much more secure attachment because they’re able to go away and not be distressed*,* or whatever the worries were around attachment styles. You’d ideally have some of that*,* but that’s really hard to do” (ID: P8).*

There were specific barriers and enablers in relation to commissioning parent-infant relationships services:

#### Importance of infant mental health

Although many commissioners were aware of the need to consider babies’ mental health and the importance of PAIR, some mentioned that they did not think about the mental health of those under the age of 5 years.

*“They [clinicians] will do assessments for under-5s*,* but from a sort of an emotional pathway point of view*,* I guess it’s never*,* there’s never been evidence that the need is there. I guess there’s never been something that’s been said or there’s a cohort of young people that we need to provide a provision for…It would be more of a*,* you know*,* if there was a really clear identified physical disability or learning disability or something. It’s never*,* you don’t tend to get notifications in relation to mental health for under-5s” (ID: P2).*

There was also some confusion about language and concepts relating to the topic of mental health in infancy and who the service/support was for:

*“I mean when you’re talking about sort of like attachment*,* uhm*,* obviously there are services that do look at that*,* and I suppose I was*,* I’m aware of those*,* it’s just I wasn’t*,* I wasn’t thinking about specific infant mental health as in*,* it’s more I was thinking more of these like obviously the sort of uhm*,* the work being done with the mother rather than thinking about actually inputting with the child*,* yeah” (ID: P2).*

Further, unless they were aware of specialist PAIR services, most commissioners could only think of health visitors as a key professional who would be involved in an infant’s life. They were therefore unaware of potential solutions to PAIR difficulties and what different levels of care could be commissioned. It was acknowledged that services should be provided for mental health in the under 2 s and that there were gaps in provision, in part due to a lack of dedicated commissioning of services specific to infant mental health. It was also raised by commissioners that investing in babies’ mental health at an early stage may help to prevent problems later in childhood and adulthood.

*“If we’re really going to be honest about infants*,* other than the role of the health visitor*,* who’s looking out for the infants? Who’s got the infants in mind? And you know they’re our next generation*,* if we’re going to invest anywhere*,* that’s where we should be investing into” (ID: P1).*

However, there were some reported difficulties in knowing how to access evidence about the impact of PAIR on children’s outcomes and knowing where to find evaluations of PAIR services, and these were barriers to commissioning support.

Those commissioners who were aware of baby brain development and the evidence about the critical period of development in infancy felt it was important that there should be more awareness and sharing of this type of information both to professionals and also the public. It was suggested that if more commissioners working in the field of perinatal/mental health/child services were aware of the impact of infant mental health, then this would be an enabler to commissioning.

*“Uhm. I guess it is just*,* it’s that evidence base isn’t it? So that’s sort of again like*,* it’s about how the profile is raised…About sort of infant mental health. And actually*,* I guess some sort of like*,* yeah*,* yeah*,* just sort of raising the profile in the evidence*,* really…I would want to see it as part of a comprehensive pathway. Uhm and fully costed up with the right workforce” (ID: P3).*

#### Maternal and parent-infant services/support

Commissioners acknowledged that family support for those with new babies/children was essential and that support for bonding/attachment should be available. The commissioners who were aware of specialist PAIR services, however, had some concerns about the sustainability of these types of service and the limited number of families it may be relevant to. These issues were perceived as barriers to commissioning. It was reported that perinatal/maternal/health visitor practitioners could be trained to support families with attachment/bonding. That is, that family support should be a whole systems approach which would mean more families could access support. This early intervention/support may remove the need for more costly specialised services.

*“So I think there is*,* there is something about early intervention in relation to attachment and trauma. Uhm. But it’s working with the system*,* isn’t it? So it’s not*,* it’s not just from a health point of view*,* it’s sort of like a system approach to how we*,* you know*,* we work together in relation to that delivery. And how social*,* social workers maybe? And I don’t know what their training is like in relation to when they’re looking at safeguarding about what they*,* what they know about sort of attachment and trauma as well. From a social care point of view” (ID: P2).*

There were some barriers and enablers related specifically to the timing of the interviews:

#### Impact of Covid-19 on commissioning

As with most things, Covid-19 had an impact on how services were commissioned and delivered. It was reported that the pandemic had also put more pressure on the NHS and mental health services. In relation to infant mental health and PAIR, it was noted that many services such as health visitors had moved online during the pandemic, with practitioners communicating with families using remote methods. There was concern that some areas were still using remote methods, which may have had a negative effect on families. Therefore, commissioners felt that during the pandemic demand for PAIR services may have increased and that the services that support families were under more pressure and/or had experienced changes, therefore compounding the issue. Also, the pandemic had created a backlog for referrals, and this was creating more demand and pressure on the services available such as maternal and mental health services which were picking up PAIR difficulties due to a lack of commissioned PAIR services.

There were also some perceived benefits of the Covid-19 pandemic reported, with some commissioners indicating that they had to be more responsive during the pandemic and the process of setting up services was sometimes quicker, and therefore more responsive to population need.

*“So I think commissioning is changing. Uhm*,* I think during the pandemic it became easier in a way. Uhm*,* because a lot of the processes were just– not stood down– they became quicker and a bit more*,* let’s respond now*,* instead of*,* instead of working it out and making sure it’s 100%*,* let’s go on a gamble. And if we 75% think it’s right*,* let’s just do it and see if it works. And it was a lot more*,* let’s pilot and test and learn as we go. And I’ve always said I think the NHS has a*,* has a fear of failure*,* and I get that completely because failure ultimately could lead to death. But actually*,* we don’t embrace failure enough*,* that the try*,* fail*,* try again*,* fail*,* try better approach does not work in commissioning. If it fails*,* it’s decommissioned. If it succeeds*,* we go around the country telling everyone ‘aren’t we wonderful?’ There is no middle ground. And I think the pandemic allowed us to have some of that middle ground of*,* this little bit of it worked*,* but actually the rest of it didn’t” (ID: P5).*

In addition, online parent groups which were set up during the pandemic had been well attended with some dads also joining which was reported as a change from the norm:

*“ And the other thing we’re starting to see just a little bit is more dads engaging in that resource. Uhm*,* ‘cause [sic] historically it’s always been mum*,* with a couple of exceptions*,* but generally*,* generally speaking” (ID: P4).*

This is significant because infants also form relationships with dads which are important to their development and the online groups had given the opportunity to support this relationship in a way that they had not in the past. It indicated to commissioners’ the types of services that they could commission if they wanted to be inclusive of fathers.

The findings highlight potential gaps in knowledge about and implementation of PAIR services and the importance of considering the mental health of infants. Identifying how and where commissioning behaviours can realistically be changed is the next key step.

#### Changing behaviours

The barriers and enablers identified from the interviews are summarised (Tables 1 and 2) and those at the individual level are mapped on to the COM-B model. There were some barriers and enablers which were identified that were beyond the level of the individual behaviour change and therefore out of scope for this work which only aimed to influence individual level levers.

The behaviour of interest is commissioning PAIR services. Capability is defined as the individual’s psychological and physical capacity to engage in commissioning PAIR services. Opportunity is all the factors that lie outside the individual that make PAIR commissioning possible or prompt it. Motivation is the brain processes that energise and direct behaviour, including habitual processes, emotional responding and analytical decision-making.

**Table 1:** Barriers to commissioning

Theme	Issue	COM-B
The commissioning process	N/A	N/A
Collaborative approach to commissioning	N/A	N/A
Funding process	Some local issues not in national priorities and therefore not funded	N/A
	Short term funding	N/A
	Funding uncertainty can make recruitment difficult– therefore shortage of skilled workforce	N/A
Service impact and evaluation	Challenging to demonstrate impact and value for money	Capability
Importance of infant mental health	Many commissioners did not think about mental health of infants	Motivation
	Commissioners not able to think about who could support infant mental health or parent-infant relationships	Capability
	Lack of dedicated commissioning	N/A
Maternal and parent-infant services/support	Concerns regarding sustainability of parent-infant relationships services	N/A
	Concerns about numbers of families impacted by difficulties and need for specialist services	Motivation
Impact of Covid-19 on commissioning	Increase in demand for parent-infant services due to pandemic	Motivation

**Table 2:** Facilitators to commissioning

Theme	Issue	COM-B
The commissioning process	Striving to reducing health inequalities from early life	Motivation
	Change from CCGs to ICSs	N/A
	Sharing of information between commissioners	Opportunity
Collaborative approach to commissioning	Understanding population needs	Capability
	Ability to engage families in commissioning	Capability
	Open and honest relationship with providers	Opportunity
Funding process	N/A	N/A
Service impact and evaluation	N/A	N/A
Importance of infant mental health	Understanding importance of early investment to save later	Capability
	Understanding the evidence around baby brain development	Capability
	Need for more commissioners working in this area	N/A
Maternal and parent-infant services/support	Recognising importance of family support for new parents	Motivation
	Whole systems approach needed	N/A
Impact of Covid-19 on commissioning	Quicker response times	Opportunity

Using the COM-B components, the individual-level facilitators and barriers to commissioning PAIR services as identified in the commissioner interview findings, can be mapped onto the model. This provides a behavioural diagnosis of what potentially needs to be changed to enable the commissioning of such services (Table 3). The need for change was then established and, as Table 3 shows, there was a need for change across all areas to support individual level commissioning behaviour. Additionally, the intervention types and policy options that could most effectively change behaviour were identified, using a process informed by West et al. [27]. Table 3 shows that common intervention types, including education and training, could address multiple issues across capability, opportunity and motivation. Further, there were also shared policy categories, such as guidelines and communications and marketing which could shape behaviours.

**Table 3:** COM-B components for target behaviours for commissioning parent-infant relationship services

Target behaviour: Commissioning PAIR services				
COM-B Components	What needs to happen for the target behaviour to occur?	Is there a need for change?	Intervention function	Policy category
*Capability*	Understanding of population needs for parent-infant relationship services Ability to engage families in commissioning Understanding of the evidence relating to baby brain development Recognition of the importance of early investment resulting in later savings Understanding which parts of the workforce can support parent-infant relationships Being able to demonstrate impact and value for money of services	Where commissioners do not have the appropriate knowledge and skills, they may not have the capability to commission parent-infant relationship services that are appropriate, accessible, and effective.	Education Training Enablement	Guidelines Communications and marketing Service provision Fiscal measures
*Opportunity*	Relationships with other commissioners that support information sharing Open and honest relationships with providers	Where commissioners do not have supportive relationships with other commissioners and service providers their opportunities to commission parent-infant relationship services may be reduced.	Training Environmental restructuring Modelling Enablement	Guidelines Communications and marketing Service provision Fiscal measures
*Motivation*	Recognising the importance of family support for new parents Increased demand due to covid-19 pandemic Awareness about mental health in infancy among commissioners Information about the demand at different levels of care	Where commissioners are not aware of infant mental health and parent-infant relationships or the impact that the pandemic has had on demand they may not be motivated to commission parent-infant relationship services at different levels of care.	Education Persuasion Incentives Training Environmental restructuring Modelling Enablement	Guidelines Communications and marketing Service provision Fiscal measures

In collaboration with the Parent-Infant Foundation [28], it was proposed that a new PAIR services commissioning toolkit, with a focus on key behavioural change initiatives such as education and training, would help commissioners and their local colleagues to address barriers and foster enablers. The toolkit would build a shared understanding of the topic, help to clarify local population needs, offer tools to help map existing provision and support to understand how these gaps could be addressed through commissioning, and how to evaluate PAIR services.

## Discussion

This is the first qualitative interview study with commissioners to explore barriers and enablers to commissioning PAIR services. This study included commissioners with a wide range of portfolios relevant to PAIR and therefore their insights relate to PAIR commissioning but also their broader remit. It was acknowledged by commissioners that there was a lack of dedicated commissioning of PAIR services and instead issues relating to infant mental health were falling to other services to address. Some commissioners noted that they did not think about the mental health of infants and young children in the early years. In addition, there was some confusion around the language and concepts relating to infant mental health and for some commissioners there was a lack of clarity in relation to which parts of the workforce could provide PAIR support. However, there was a recognition that support during this period is vital for families and there was suggestion that a whole systems approach may be indicated.

Interviews revealed key actionable insights into factors affecting commissioning at the individual level and beyond. At the individual level, commissioners highlighted capability (including skills and knowledge), opportunity (including social relationships), and motivation (including emotions and beliefs) all impacted commissioning decisions. There were additional levers identified beyond the level of the individual including barriers relating to funding, including issues around national funding not reflecting local priorities and the short-term nature of funding and the impact of this on workforce retention. Using a pragmatic approach, we chose to focus on those factors within our control and identified best-fit intervention categories in line with project budget resources. We used the findings as the basis for a workshop to co-develop a commissioning support toolkit with commissioners. As in the previous literature, commissioners demonstrated using a range of information to inform their decisions [13] including family voice, insights from other commissioners, and discussions with providers. However, they highlighted challenges in using evidence from research, as seen in other studies [14], for example not understanding the science of early relationships. Previous work by Homonchuk [18] had shown that there were factors which were influencing commissioning decisions which could be addressed, and in this study, we utilised the COM-B model to diagnose specific issues that could be targeted at the individual and system level. Using the BCW it was possible to identify potential intervention types, including education and training, and policy categories, such as communications and marketing, which could address factors that would support or hinder commissioning.

Specific insights into the covid-19 pandemic and commissioning were highlighted. In particular, commissioners emphasised the impacts of the pandemic restrictions on services that would normally support parents and infants moving to remote delivery which they considered to have had negative impacts on families. This is also reflected in the literature [29] with more vulnerable families being impacted the most. This may have increased demand for PAIR services. However, despite commissioners experiencing an ability to quickly respond to population need during covid-19 their focus was not on commissioning PAIR services at this time and the increased demand for services from families was being picked up indirectly in other services such as perinatal mental health.

### Study strengths

This is the first paper to explore the key barriers and enablers of commissioning PAIR services in an area of commissioning that experiences high levels of inequalities in access. This study was able to identify actionable findings that could be addressed in a commissioning support toolkit when 75 local areas in England were receiving dedicated funding through Family Hubs and Start for Life. In addition, this work provides insights which are transferable to other areas of commissioning due to the broad portfolios of responsibility of the commissioners who were interviewed.

### Study limitations

Commissioners were recruited from the North East North Cumbria region only, although commissioners from other areas were involved in a practice involvement group which informed the design and delivery of the work. Future studies could interview commissioners from other regions within the new commissioning arrangements.

### Implications for policy/practice/research

This study highlighted the need for a practical resource that could address individual level barriers and enablers to commissioning PAIR services. The findings were used to inform the development of a co-produced commissioning support toolkit, including key behaviour change intervention types, which is now hosted for free on the Parent Infant Foundation website [30]. However, this work also identified barriers and enablers beyond the level of the individual commissioner at a more systems level, particularly those relating to funding which we were not able to address. The recent Family Hubs and Start for Life funding for perinatal mental health and PAIR has gone some ways to addressing this for selected local areas. However, key barriers such as uncertainty around funding sustainability remain. In addition, funding intersects with issues relating to workforce. PAIR services require a skilled workforce, but funding uncertainty may result in skill gaps when professionals seek roles with greater employment security.

The move to ICSs and the Family Hubs and Start for Life focus on more integrated services and pathways which have followed, will to some extent, address the issues highlighted here with respect to a need for more whole systems approaches to commissioning PAIR services. However, there may be ongoing challenges in relation to implementing integration which will require attention.

This study also identified some barriers and enablers to commissioning PAIRs services, which could be addressed by policy categories at a national level. These included communications and marketing to educate or persuade individuals about the importance of parent-infant relationships. Department of Health and Social Care (DHSC) campaigns such as If They Could Tell You [31] including social media posts and posters highlight the value of parent-infant relationships to families. Similar campaigns could address the needs of commissioning audiences, for example Parent-Infant Foundation’s Infant Mental Health Awareness Week [32] and the recent Speak Up For Babies campaign from First 1001 Days Movement [33]. In addition to this, commissioners indicated that a move to a whole systems approach may be beneficial, allowing more families to access support and this is reflected in Family Hubs and Start for Life programme [9] emphasising providing more joined up services.

### Next steps and further research

The findings from this study were shared at an online workshop with commissioners from across the North East and Cumbria and they were involved in the co-production of the commissioning toolkit [30], steering content and design principles. Further research could evaluate the utility of the co-produced toolkit in addressing the barriers and fostering the enablers and whether providing education and training materials has been sufficient to change commissioning behaviours.

## Conclusions

This is the first study to map the barriers to and enablers of PAIR service commissioning in England. Through understanding individual and system factors impacting commissioning PAIR services, we can identify promising behaviour change initiatives to actively mobilise evidence to enable commissioners to increase access, improve outcomes, and decrease disparities and address infants as an under-served group.

## Supplementary Information

Below is the link to the electronic supplementary material.

Supplementary Material 1

Supplementary Material 2

## Data Availability

Data can be made available on request from the corresponding author. The datasets generated and analysed during the current study are not yet publicly available due to privacy provisions but can be made from the corresponding author on approval from the authorising ethics committee.

## Abbreviations

PAIR: Parent-Infant Relationship
ICS: Integrated Care System
COM-B: Capability Opportunity Motivation-Behaviour
BCW: Behaviour Change Wheel
CCG: Clinical Commissioning Group
