# Supplementary material for: “Who’s got the infants in mind?” A qualitative exploration of the barriers and enablers to commissioning parent-infant relationship services in England
Source: BMC Health Serv Res. 2025 Aug 2;25:1020. doi: 10.1186/s12913-025-13215-5 (PMC12318414; doi:10.1186/s12913-025-13215-5)
Supplement: Supplementary file 1 — Supplementary Material 1 [file 12913_2025_13215_MOESM1_ESM.docx]

**Commissioning Infant Mental Health Services**

**Commissioner/stakeholder Interviews**

**Topic Guide**

**Introduction**

Thank you for agreeing to talk to me today. As outlined in the study information sheet, we are going to talk about service transformation for babies and their families to inform the development of a tool to facilitate the commissioning process and to determine how such a tool could help services reach more families and reduce inequalities.

The interview should take between 45 minutes to an hour and you are free to stop the interview at any time if you decide you no longer wish to take part. Can I check you’ve read the information sheet and you’re happy to proceed?

***Confirm e-consent has been completed***

***Switch on voice recorder***

1. Can you please tell me a little about your role as a commissioner?
   - Time in post
2. Where work/ed
   - Which services/areas they focus on
   - Which future outcomes are they most interested in?
     - Health changes in their local population?
     - Economic benefits?
     - Reducing burden of the workforce?
3. Do you feel the commissioning process has changed/is changing?
   - What do you feel are the important changes?
   - What do you feel this does for your role?
4. How important are national government documents or guidance when it comes to you making decisions about commissioning?
   - What guidance used?
     - NHS long term plan?
     - Others?
   - In relation to maternal and child health
   - In relation to mental health services
5. Have you heard the term infant mental health before?
   - What does it mean to you?
   - What do you think it involves in practice?
6. Can you tell me about your experiences/knowledge of infant mental health?
   - Have you commissioned such services in the past?
     - *Some people may talk about universal services or specialist services (be aware of differences – specialist needs a trained therapist/ psychotherapist)*
   - Do you commission them now?
   - Have you ever decommissioned such services?
     - *If yes,* ask why
   - *If during the interview, the interviewee appears focused on perinatal/adult services ask the following:*
   - Do you think there are any differences between perinatal and infant mental health services?
   - How relevant is the mental health of babies is to your current priorities?
7. ***If interviewee answers ‘yes’ to commissioning IMHS:***
   - What would you say facilitates the commissioning of such services?
   - What helped you to make the decision about commissioning these services?
   - Did you liaise with any other IMH commissioners or IMH teams across the UK when commissioning?
     - To find out how other areas work?
     - To collect best practice evidence?
     - To find out how they are funded?
   - Can you explain how the commissioning process works for your local IMHS?
     - What, if any, barriers did/do you face and how did you overcome them?
     - What population was the service commissioned for?
       - What change do you want to see in this population?
     - How were the needs in your locality assessed?
     - Who is involved in the commissioning of IMHS?
       - Families
       - Practitioners (Midwives/Health Visitors
       - IMHS champions
       - Academics or other experts
       - Others?
     - How does the service report its impact to you?
       - Do they use any particular evaluation tools or methods?
       - What type of information or data from them do you find most compelling or informative?
     - Can you tell me where the funding for such services comes from?
       - Is this short/long term?
       - Temporary/permanent?
     - Do you feel the IMHS has adequate reach?
     - How relevant is the issue of family inequalities to the commissioning of the local IMHS?
       - Is there anything in the commissioning service specification and contract specifically about inequalities?
       - How does the IMH team address inequalities?
       - How does the IMH team measure its impact on inequalities?
     - Have you been able to involve any practitioners in the commissioning process and if so who and how?
     - Did you consider data when making your decision to commission IMHS?
       - What data did you use?
       - What was helpful about the data?
     - Did you consider evidence relating to cost-effectiveness?
     - To what extent do storytelling and the voices of practitioners influence your commissioning decisions?
     - To what extent do storytelling and the voices of families influence your commissioning decisions?
     - What do you think are the 3 most important pieces or types of information that affect the commissioning of IMH services?
   - *Recap what’s been discussed and ask interviewee to expand on answers if/as appropriate*
8. ***If interviewee answers ‘no’ to commissioning of IMHS:***
   - Can you tell me more about why specialised IMHS services have not been commissioned in your locality yet?
     - *Make sure they are aware of the differences between universal and specialist services*
   - Have you considered commissioning these services?
   - *If interviewee specifies that they have a perinatal MH team that offers parent-infant support, then ask:*
     - Are there any babies facing IMH difficulties that the perinatal service can’t see?
     - Are there any babies whose parents wouldn’t qualify for perinatal services but who face IMH difficulties?
   - How relevant is the issue of family inequalities to the commissioning of the local IMHS?
     - Is there anything in the commissioning service specification and contract specifically about inequalities?
     - How does the IMH team address inequalities?
     - How does the IMH team measure its impact on inequalities?
   - What if anything, do you think is preventing the commissioning of IMHS?
   - What do you see as the barriers to decision making?
   - What kind of things do you think about when considering commissioning such services?
   - How are the needs in your locality assessed?
   - Where would funding for such services come from?
   - Have you seen any evidence or data about IMH or baby brain development?
     - What did you think about this evidence?
     - Are there any ways in which this evidence could be improved?
   - How often is scientific research about babies generally used in commissioning do you think?
     - How relevant is scientific research about baby’s brains or IMH to your personal commissioning decisions?
   - What role does cost-effectiveness play in decision making around commissioning IMHS?
   - Who do you think should be involved in the commissioning of IMHS?
     - Families
     - Practitioners such as:
       - Midwives
       - Health visitors
     - IMH Champions
     - Academics or other experts
       - Others?
   - How can the commissioning of IMHS be facilitated?
     - What support is required?
     - What data would be helpful?
     - What information about cost-effectiveness would be useful?
     - How do you feel about visiting/liaising with other CCGs and/or LAs?
       - To find out how other areas work
       - To collect best practice evidence
       - To find out how they are funded
     - To what extent do storytelling and the voices of practitioners influence your commissioning decisions?
     - To what extent do storytelling and the voices of families influence your commissioning decisions?
     - Do you think this type of data would be useful?
   - What do you think are the 3 most important pieces or types of information that affect the commissioning of IMH services?
   - *Recap what’s been discussed and ask interviewee to expand on answers if/as appropriate*
9. Lastly, to what extent do you believe adults are influenced by their experiences as a baby?
10. Thank you for sharing your experiences with me today. Is there anything else you would like to add?

***Switch off voice recorder***
